# Unilateral Lumbo‐Pelvic Fixation for Denis Type I Unilateral Sacral Fracture: A Finite Element Analysis

**DOI:** 10.1002/jsp2.70022

**Published:** 2025-01-20

**Authors:** Jie Yang, Kai O. Böker, Xishan Li, Xiang Zhou, Wolfgang Lehmann

**Affiliations:** ^1^ Department of Trauma Surgery, Orthopaedics and Plastic Surgery University Medical Center Göttingen Göttingen Germany

**Keywords:** bilateral lumbopelvic fixation, finite element analysis, pelvic fracture, triangular osteosynthesis, unilateral lumbopelvic fixation

## Abstract

**Background:**

Unilateral sacral fractures with posterior ring instability represent a prevalent type of posterior pelvic ring fracture. While lumbo‐pelvic fixation is recognized as a highly stable method, the sufficiency of unilateral lumbo‐pelvic fixation (ULF) for such fractures remains under debate.

**Purpose:**

This study aims to assess the biomechanical stability of ULF compared to traditional bilateral lumbo‐pelvic fixation (BLF) and triangular osteosynthesis (TO), incorporating clinical observations, and previous biomechanical data.

**Methods:**

We developed a three‐dimensional spine‐pelvis model to simulate a unilateral sacral fracture with posterior ring instability. The model was used to compare the stability of ULF with BLF and TO, utilizing both newly generated data and ULF models reported in existing literature.

**Results:**

Our findings indicate that BLF and TO provide greater stability than ULF, with BLF emerging as the most stable model. While ULF may be insufficient for immediate postoperative weight‐bearing, TO also demonstrated potential risks of instability during rotational and lateral bending movements toward the fracture side.

**Conclusion:**

Despite its application in clinical settings, ULF may not adequately support early postoperative mobility. This study underscores the need for cautious application of ULF and suggests that enhancements such as additional fixation points may be necessary. The results also highlight the importance of tailored postoperative rehabilitation strategies for patients undergoing TO, especially in managing movements that could destabilize the fracture site.

## Graphical abstract

The article investigates the effectiveness of unilateral lumbo‐pelvic fixation (ULF) for treating unilateral sacral fractures compared to traditional bilateral lumbo‐pelvic fixation (BLF) and triangular osteosynthesis (TO). Using finite element analysis, the study finds that while ULF can offer some stability, it is generally less stable than BLF and TO, especially under certain movements. The research highlights the potential risks associated with ULF and suggests that more robust methods like BLF and TO are preferable for ensuring post‐operative stability and promoting optimal healing.

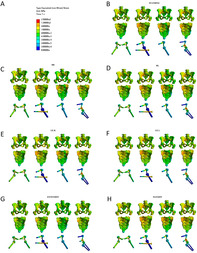

## 1 INTRODUCTION

The sacrum is formed by the fusion of five sacral vertebrae, containing the cauda equina within the sacral canal, sacral nerves through the anterior and posterior sacral foramina, and is surrounded by many nerves, blood vessels, and pelvic organs. In terms of gravity and stress conduction, the L5/S1 intervertebral disc accounts for about 80% of the biomechanics of the lumbosacral region [1]. As part of the posterior pelvic ring, the sacroiliac joint complex is responsible for 60% of the overall stability of the pelvis [2]. In cases of high‐energy trauma, fractures of the sacrum are a critical factor to consider, especially given their connection to injuries in the pelvic ring, which plays a significant role in maintaining the stability of the pelvis [3].

The surgical treatment of unstable fractures in the posterior pelvic ring is now widely accepted as the standard approach. Various methods of internal fixation are employed, including the use of locking compression plates, reconstruction plates, sacroiliac screws, and pedicle screw‐rod systems [4, 5, 6, 7]. Lumbo‐pelvic fixation has been identified as the biomechanically most stable method for treating posterior pelvic ring injuries. This technique involves the surgical connection of the fractured sacrum with the ilium to the spine, enhancing stability and promoting optimal healing conditions. [8, 9, 10] This technique first introduced by Käch and Trentz [11] has been further refined by Schildhauer et al. [9] They enhanced the method by incorporating sacroiliac screws, leading to the development of “Triangular osteosynthesis.” This advancement is recognized for its exceptional stability in treating injuries to the posterior pelvic ring, marking a significant evolution in the surgical approach to these complex fractures [12]. However, this treatment requires a certain amount of experience in spinal surgery. Even if it is biomechanically the most favorable, therefore some pelvic surgeons do not use it or only together with spine surgeons. But it is a fundamentally simple technique that basically belongs in the repertoire of a pelvic surgeon.

**FIGURE 1:**
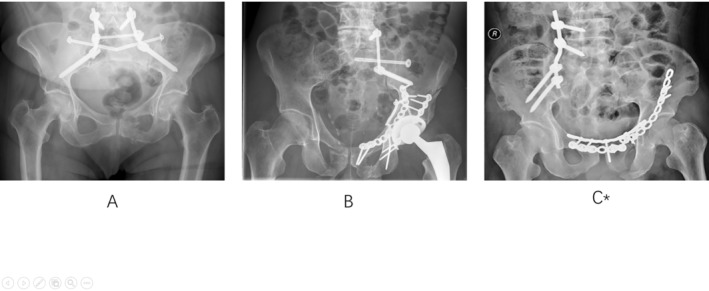
(A) Bilateral lumbo‐pelvic fixation (BLF); (B) triangular osteosynthesis (TO); (C) unilateral lumbo‐pelvic fixation (ULF); * reprinted from injury, 52(8), Qiao, B., Liu, J., Ni, W., Shui, W., Luo, G., & Guo, S, unilateral lumbopelvic fixation for AO/OTA Type C1 and C2 pelvic fractures: Clinical efficacy and preliminary experiences in 23 patients, 2333–2338, Copyright (2021), with permission from Elsevier.

Significantly, among Type C pelvic fractures (AO/OTA), those with unilateral vertical instability, specifically Types C1 and C2, comprise 75%–78.8% of cases, with the majority being unilateral sacral fractures (USF) [13, 14]. For these unilateral sacral fractures, the traditional approach involves bilateral lumbo‐pelvic fixation (BLF) and triangular osteosynthesis (TO). With advancements in surgical techniques and internal fixation tools, some orthopedic surgeons now hold the view that unilateral lumbo‐pelvic fixation (ULF) can offer ample stability [15] (Three clinical case images shows the different fixation methods as a reference Figure 1). If this is indeed the case, then this new fixation method undoubtedly has the potential to reduce surgical time and costs, while also being less invasive. This aligns well with the current trend toward “minimally invasive surgery (MIS).” Biomechanical research in this area is still relatively uncommon. This study aims to investigate, through finite element analysis, whether ULF can provide sufficient stability for USF.

## 2 RESEARCH METHOD

### 2.1 Model Construction

This study was approved by the Medical Ethics Committee of University Medical Center Göttingen. A healthy male volunteer provided CT scans of his lumbar spine, pelvis, and femur. The images, in DICOM format, were processed in 3D Slicer 5.4.0 to extract a lumbopelvic model, which was further refined in Geomagic Wrap 2021. The model was then utilized in SolidWorks 2018 for fracture simulation and internal fixation design. Material properties were assigned to each component in ANSYS 2021R2, facilitating various analyses and comparisons.

**FIGURE 2:**
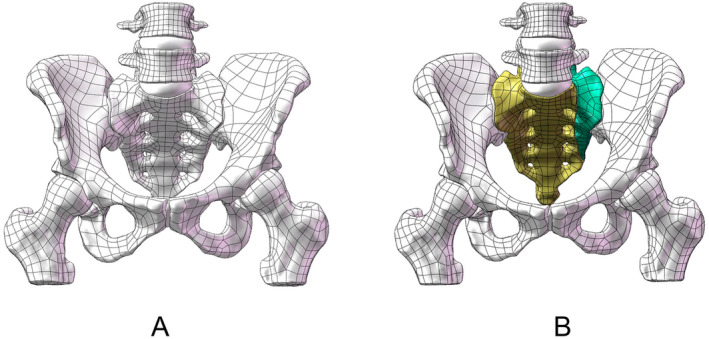
(A) Normal lumbar spine‐pelvis‐femur model and (B) unilateral sacral fracture mode.

We first created a normal lumbar spine‐pelvis‐femur model using the CT data, which was imported into SolidWorks 2018 (Figure 2A). A unilateral sacral fracture (USF, AO C1.3) was simulated by longitudinally splitting (introduced a gap of 0.5 mm at the fracture ends) the left side of the sacrum (Figure 2B). To eliminate the influence of anterior ring fixation methods on the results, this study assumes that the anterior ring injuries in all models have been firmly fixed.

In SolidWorks 2018, we modeled the implants using structural steel. The lumbar pedicle screws (45 mm length, 6.5 mm diameter), iliac screws (70 mm length, 7.5 mm diameter), and sacroiliac screws (70 mm length, 7.3 mm diameter) were crafted and their properties assigned in ANSYS 2021R2. Standard surgical techniques were applied for screw placement in the USF model. The threads of pedicle and iliac screws were omitted to simplify the models, which comprised 921 974 elements and 1 387 143 nodes. The implant element and node counts were 31 227 and 49 971 for 2‐screw ULF, 63824 and 102 197 for 4‐screw ULF, 58231 and 93 178 for TO, and 78 423 and 125 483 for BLF, respectively.

Mesh models for bones and screws were imported into ANSYS 2021R2, where spring‐damper elements simulated ligaments and muscles. Material and ligament parameters are detailed in Tables 1 and 2. These material properties were set based on existing research [16, 17, 18, 19].

**TABLE 1:** Material properties of finite element method (FEM) models.

Material	Elastic modulus, MPa	Poisson ratio
Cortical bone (Lumbar)	12 000	0.3
Cancellous bone (Lumbar)	345	0.2
Cortical bone (Ilium)	17 000	0.3
Cancellous bone (Ilium)	132	0.2
Cortical bone (Sacrum)	6140	0.3
Cancellous bone (Sacrum)	1400	0.3
Disc (Annulus)	8.4	0.45
Disc (Nucleus)	Mooney–Rivlin c1 = 0.12, c2 = 0.03	
Articular cartilage	100	0.3
Implants	200 000	0.3

**TABLE 2:** Model properties of ligament.

Ligaments	*K*, N/mm	Number of springs
Anterior sacroiliac	1500	30
Interspinous	3000	15
Long posterior sacroiliac	10 000	8
Short posterior sacroiliac	7500	30
Sacrospinous	8000	9
Superior pubic ligament	250	12
Arcuate pubic ligament	250	12

Our model's meshing was realized with the use of SOLID 187 elements. In this study, we applied composite material models separately to cortical and trabecular bones, assigning different material parameters to simulate the biomechanical behavior during fracture treatment. Specifically, the elastic modulus (E) and Poisson's ratio (ν) for cortical and trabecular bones were set based on actual physiological data to reflect their mechanical characteristics within the bone structure. Considering that the simulation load remained within the physiological activity range and induced only minor deformations in the bone, we utilized a linear elastic constitutive model to describe the behavior of these materials. This model, based on Hooke's Law, assumes a linear relationship between stress and strain. This modeling approach simplified the computational process while accurately capturing the fracture response under the predetermined loading conditions.

**FIGURE 3:**
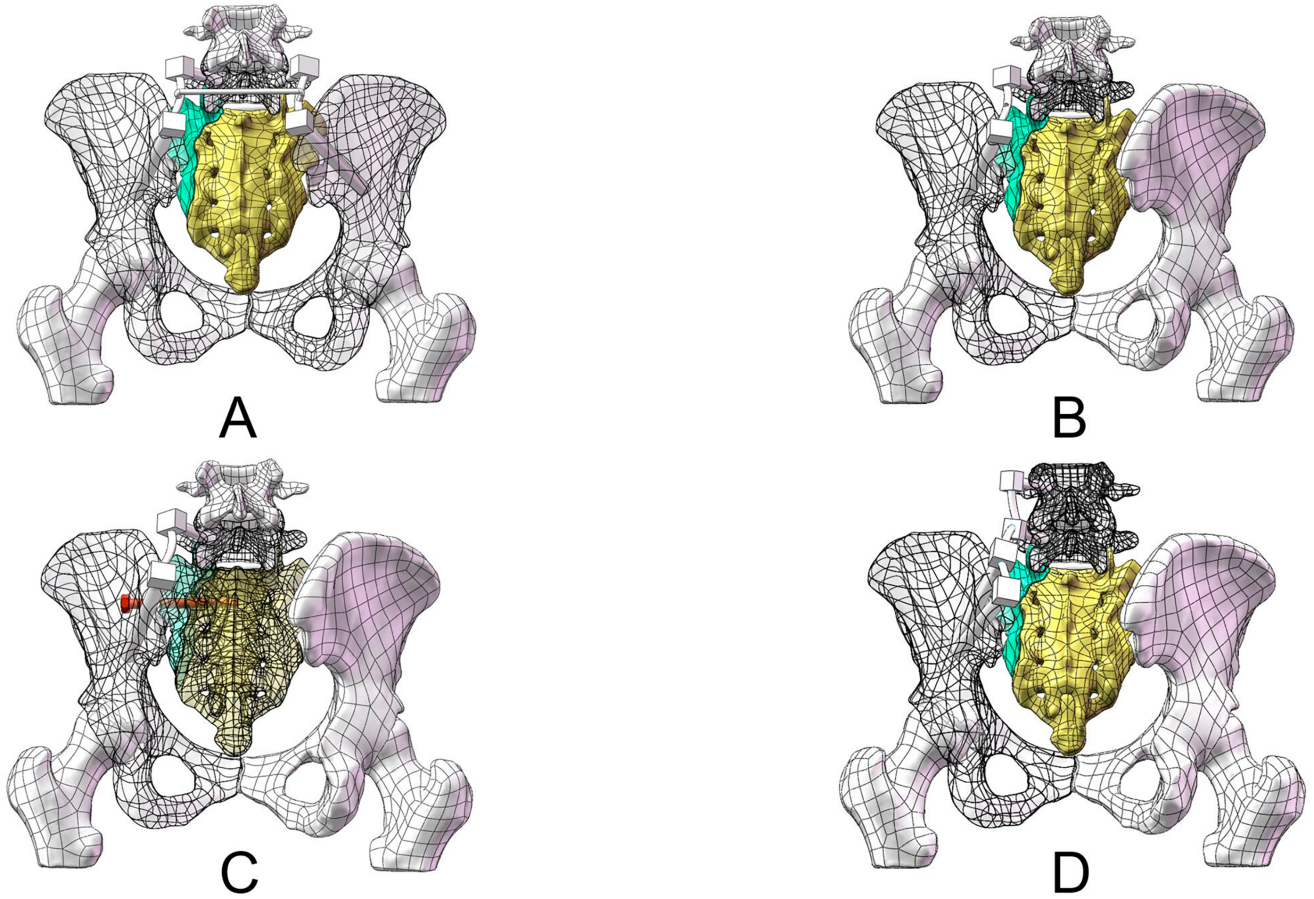
(A) Bilateral lumbo‐pelvic fixation (BLF) model; (B) unilateral lumbo‐pelvic fixation model with 2 screws (2ULF); (C) triangular osteosynthesis (TO) model; and (D) unilateral lumbo‐pelvic fixation model with 4 screws (4ULF).

**FIGURE 4:**
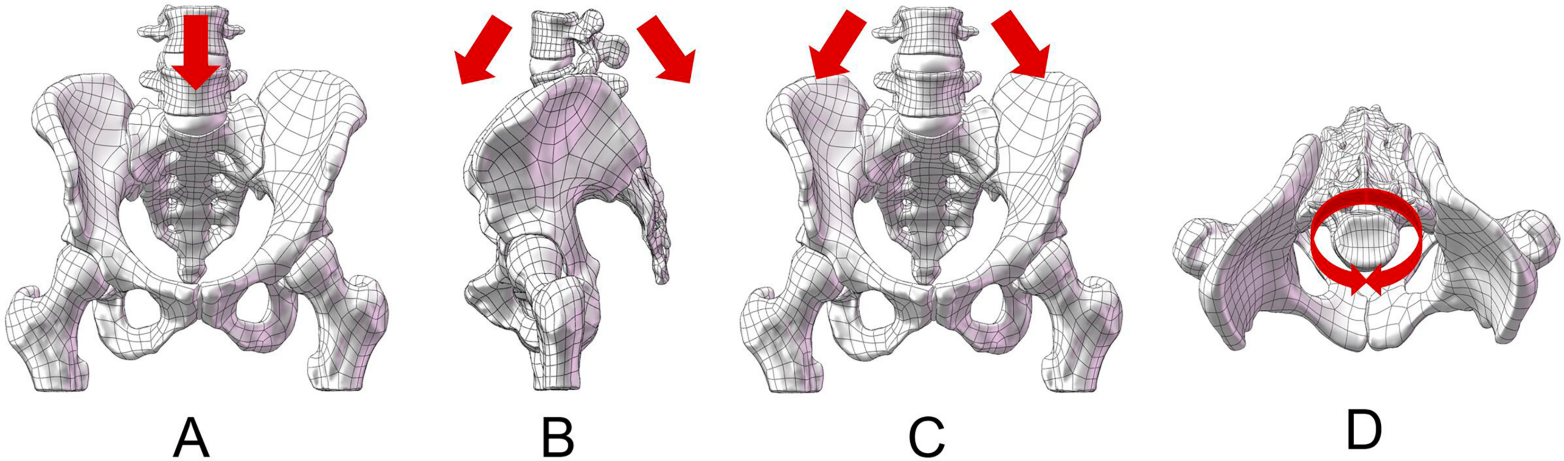
Different movements of model (A) standing; (B) flexion and extension; (C) lateral flexion; and (D) rotation.

**FIGURE 5:**
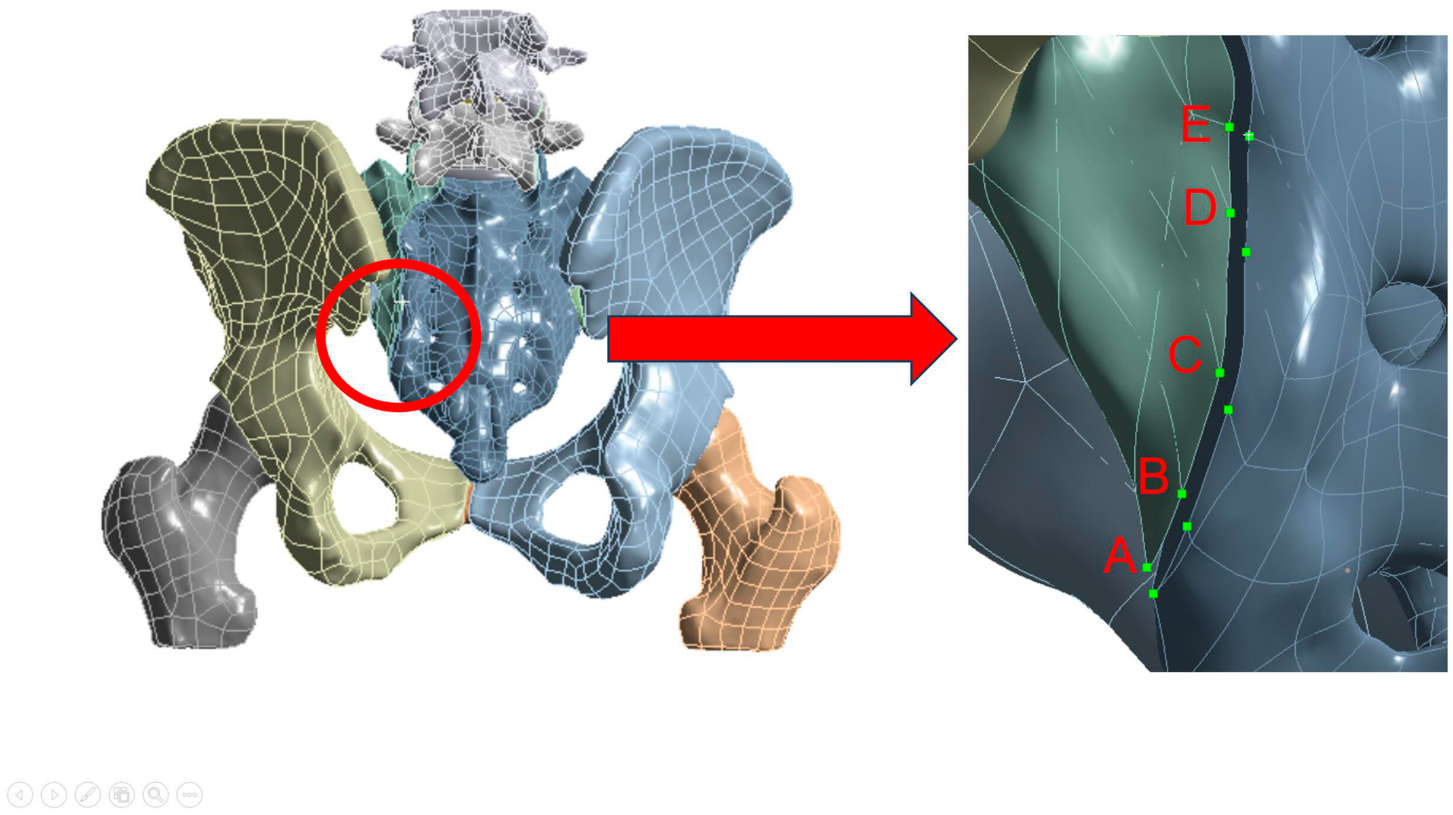
Methods for measuring displace.

Our study developed four models: 2ULF: unilateral lumbo‐pelvic fixation from L5 to the ilium (Including one iliac screws); 4ULF: unilateral lumbo‐pelvic fixation from L4‐L5 to the ilium (Including two iliac screws); TO: unilateral lumbo‐pelvic fixation from L5 to the ilium and Sacral iliac screw fixation at the S1 level; BLF: bilateral lumbo‐pelvic fixation from L5 to the ilium. The appearances of the four models are displayed in Figure 3. The models, fixed to represent a standing human posture, were subjected to a 600 N downward force and a 10 N m torque at L4 to simulate various movements (Figure 4). Stress distribution, fracture displacement, and maximum displacement were analyzed using finite element analysis. During our simulation, we observed that the primary displacement of the fracture was concentrated at the tail end of the sacrum. Consequently, we selected five pairs of points (A–E) at the fracture gap located at the sacrum's tail, as generated by the software's Mesh grid division. The changes in displacement between these points were used to represent the numerical value of fracture displacement (Figure 5).

### 2.2 Model Validation

**FIGURE 6:**
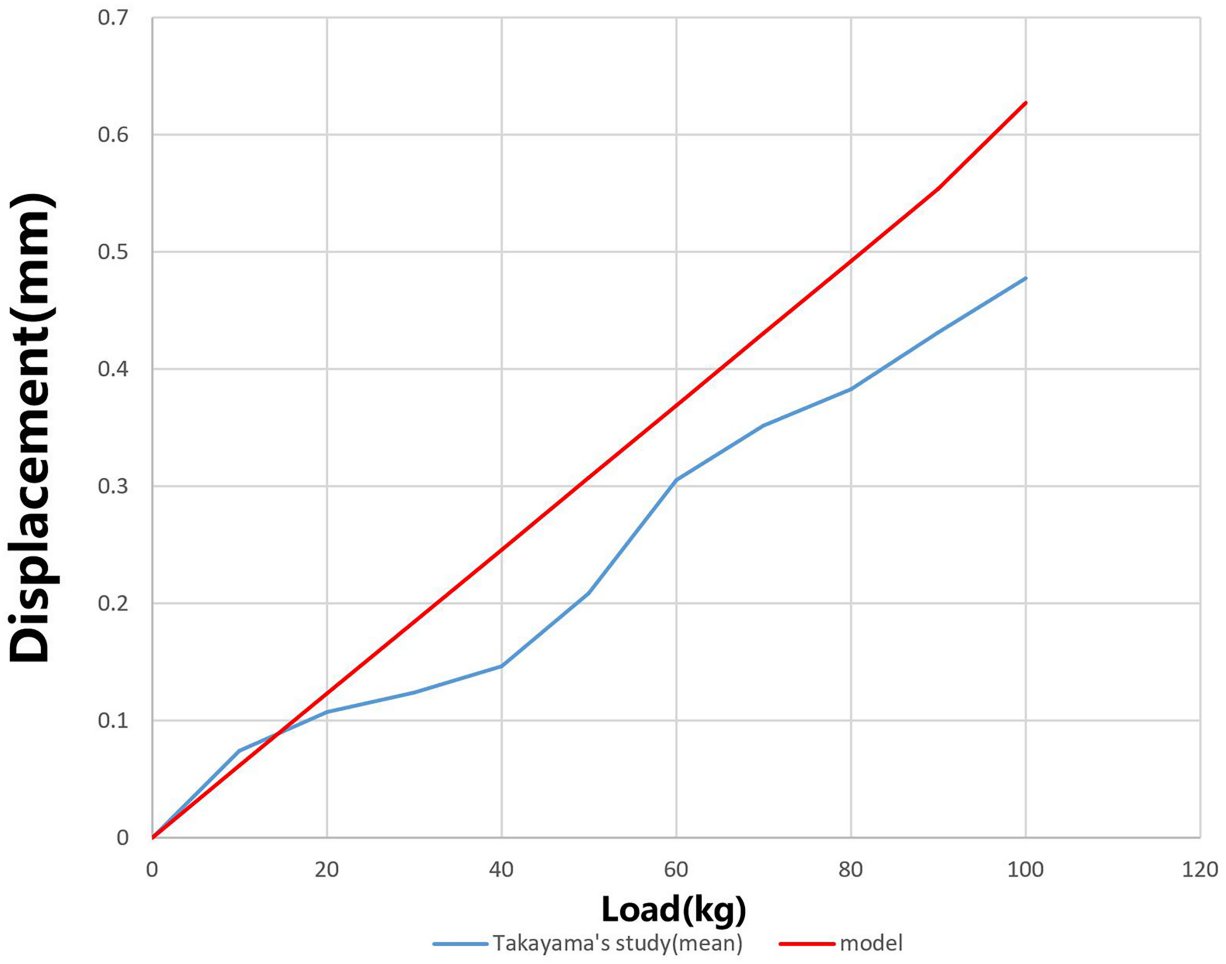
Model validation.

To ensure the accuracy and reliability of our computational models, we conducted a validation process by referencing the biomechanical experiments of Takayama [20] In this validation, we subjected our healthy lumbo‐pelvic model to the same loading tests as those performed in Takayama's study. These tests involved applying specific forces and observing the biomechanical response of the model. The resulting load–displacement curves from our computational simulations were then compared to those reported by Takayama (Figure 6). The similarity in the response curves indicated that our model accurately replicates real‐life biomechanics.

## 3 RESULTS

### 3.1 Stress Distribution

**FIGURE 7:**
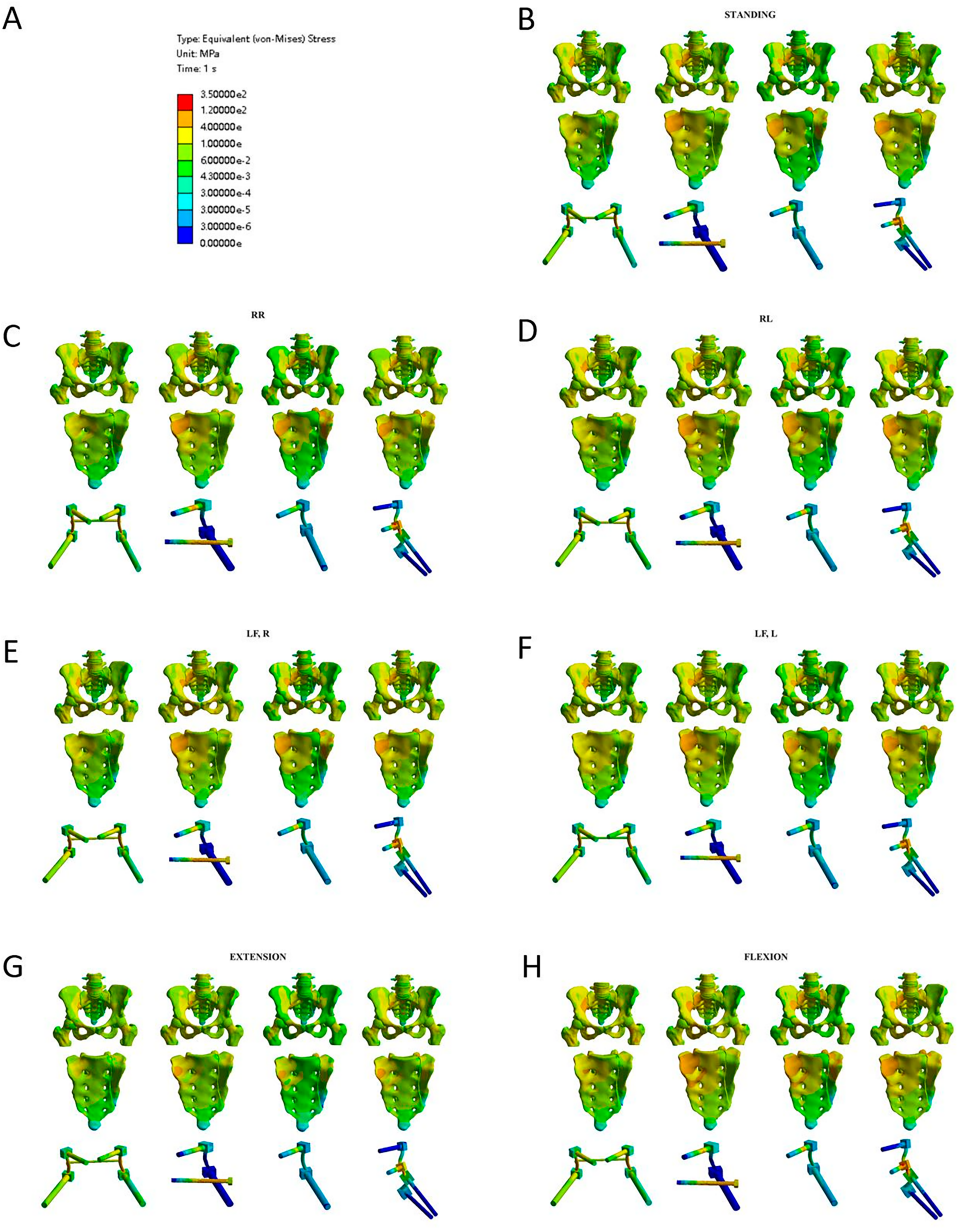
Stress distribution diagrams of four internal fixation methods under seven different postures. For each column, the figures from top to bottom represent: overall stress, sacral stress, and internal fixation stress. (A) Color scale representing von‐Mises stress distribution; (B) standing posture; (C) rotation to the right; (D) rotation to the left; (E) lateral flexion to the right; (F) lateral flexion to the left; (G) extension; and (H) flexion.

**FIGURE 8:**
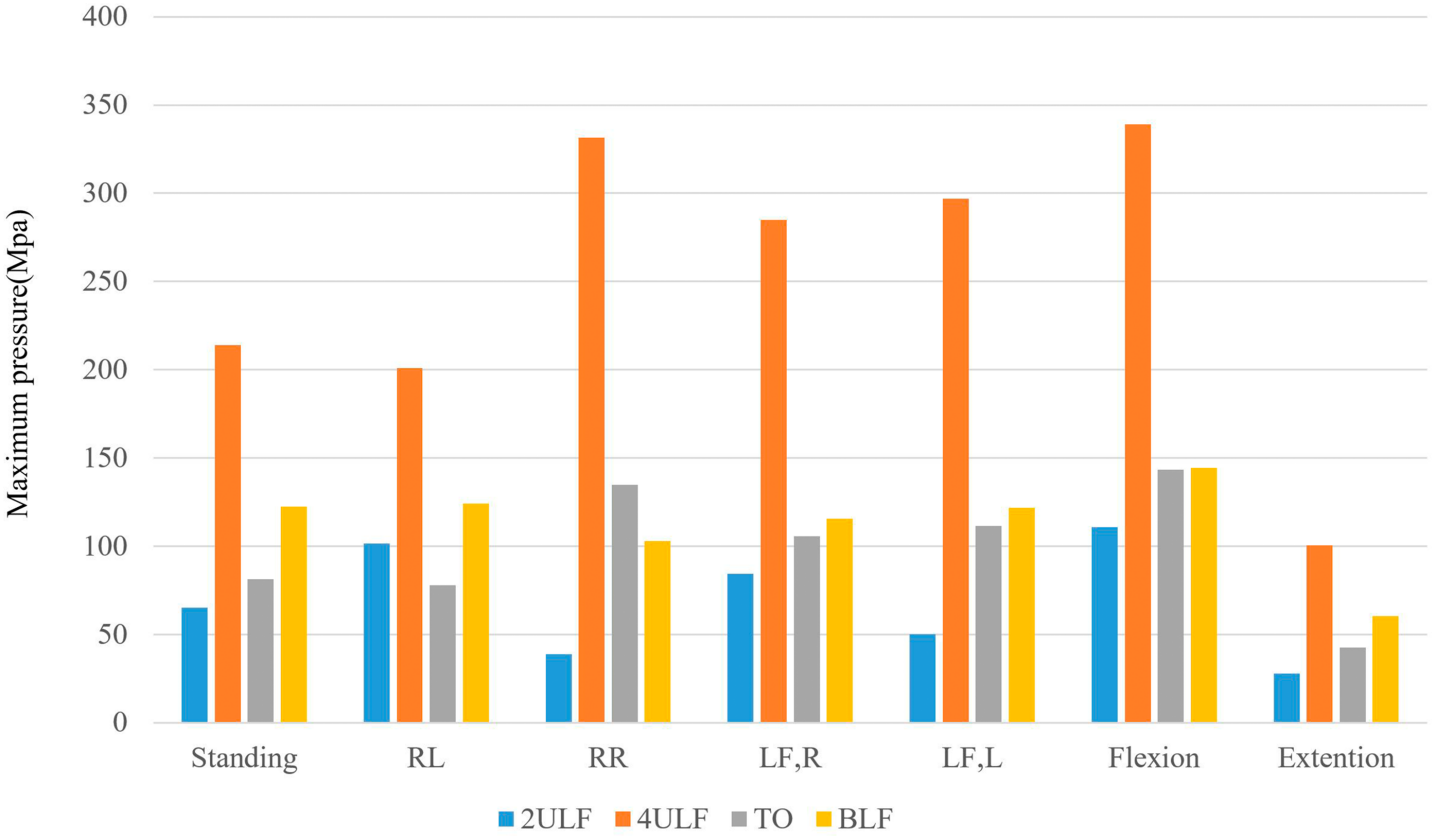
Maximum stress of four models.

The stress distribution of the 2ULF, 4ULF, TO, and BLF models are shown in Table 3 and Figure 7B–H. For the four types of internal fixation, the position with the highest internal fixation stress was flexion, while the position with the lowest was extension. The fixation method with the highest internal stress was 4ULF, with a maximum value of 338.96 MPa, as shown in Figure 8.

**TABLE 3:** Fracture displacement in different postures (mm).

Mode, point	Standing	RR	RL	LF, R	LF, L	Extension	Flexion
2ULF, A	1.44991	1.15776	1.74201	1.3813	1.51846	0.99296	1.90684
2ULF, B	1.37827	1.08847	1.66807	1.31285	1.44371	0.94935	1.80722
2ULF, C	1.24827	0.97497	1.52154	1.18502	1.31152	0.86434	1.6322
2ULF, D	1.06142	0.82481	1.29803	0.9996	1.12324	0.73685	1.386
2ULF, E	0.92923	0.717315	1.14113	0.864508	0.99393	0.64968	1.20876
4ULF, A	0.8162	0.66306	1.03576	0.529722	1.10267	0.75405	0.87833
4ULF, B	0.78627	0.56564	1.00689	0.517138	1.05539	0.72619	0.84634
4ULF, C	0.71985	0.51172	0.92797	0.47462	0.96507	0.66392	0.77577
4ULF, D	0.61768	0.44158	0.79379	0.40181	0.83355	0.56636	0.669211
4ULF, E	0.543428	0.38757	0.69928	0.34336	0.74349	0.49957	0.58728
BLF, A	0.54863	0.47819	0.61909	0.52653	0.57074	0.42324	0.67403
BLF, B	0.52434	0.45234	0.58898	0.49898	0.54233	0.40311	0.6382
BLF, C	0.46475	0.4022	0.52728	0.4437	0.485784	0.36124	0.56826
BLF, D	0.38144	0.32927	0.433609	0.36157	0.401318	0.29747	0.46541
BLF, E	0.32526	0.28103	0.369483	0.3058	0.34472	0.255165	0.39535
TO, A	0.67979	0.39388	0.9657	0.40146	0.95812	0.50368	0.8559
TO, B	0.65411	0.36917	0.93905	0.38662	0.9216	0.48531	0.82291
TO, C	0.595212	0.32762	0.86281	0.34628	0.84413	0.440594	0.749821
TO, D	0.504641	0.27726	0.73202	0.28283	0.72646	0.3694992	0.63979
TO, E	0.43562	0.23314	0.63811	0.22933	0.64191	0.318113	0.55314

### 3.2 Fracture Displacement

**FIGURE 9:**
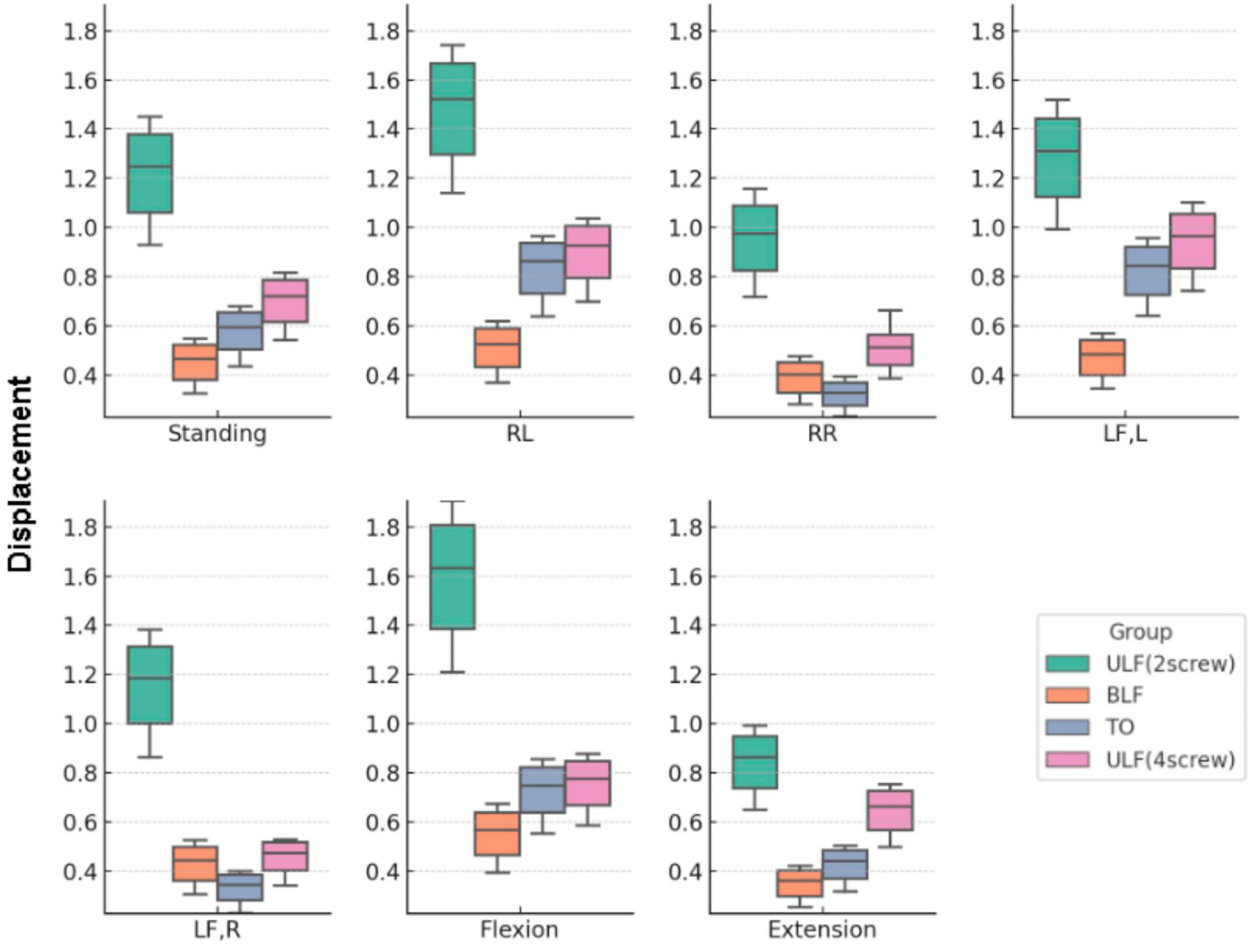
Displacement value of four models.

Fracture displacement was evaluated across all fixation models and postures, providing a measure of the mechanical stability of the constructs under various loading conditions. The results are shown in Tables 3 and 4 and Figure 9.

**TABLE 4:** Mean of fracture displacement in different postures (mm).

	2ULF	4ULF	BLF	TO
Standing	1.21342 ± 0.216902	0.696686 ± 0.114647	0.448884 ± 0.094507	0.573875 ± 0.102492
RR	0.952665 ± 0.182168	0.513914 ± 0.107378	0.388606 ± 0.082713	0.320214 ± 0.065775
RL	1.474156 ± 0.251754	0.892738 ± 0.143129	0.507688 ± 0.10487	0.827538 ± 0.13944
LF, R	1.148656 ± 0.215269	0.45333 ± 0.079228	0.427316 ± 0.09265	0.329304 ± 0.072344
LF, L	1.278172 ± 0.218547	0.940034 ± 0.150294	0.468978 ± 0.094916	0.818444 ± 0.132699
Extension	0.838626 ± 0.143874	0.642018 ± 0.107391	0.348045 ± 0.07079	0.423439 ± 0.078365
Flexion	1.588204 ± 0.289955	0.751386 ± 0.12187	0.54825 ± 0.116783	0.724312 ± 0.126621

In standing, the fracture displacements were as follows: 0.92923–1.44991 mm, with a mean of 1.21342 ± 0.216902 mm(2ULF); 0.543428–0.8162 mm, with a mean of 0.696686 ± 0.114647 (4ULF); 0.32526–0.54863 mm, with a mean of 0.448884 ± 0.094507 mm (BLF); 0.43562–0.67979 mm, with a mean of 0.573875 ± 0.102492 mm (TO).

In RR, the fracture displacements were as follows: 0.717315–1.15776 mm, with a mean of 0.952665 ± 0.182168 (2ULF); 0.38757–0.66306 mm, with a mean of 0.513914 ± 0.107378 (4ULF); 0.28103–0.47819 mm, with a mean of 0.388606 ± 0.082713 mm (BLF); 0.23314–0.39388 mm, with a mean of 0.320214 ± 0.065775 mm (TO).

In RL, the fracture displacements were as follows: 1.14113–1.74201 mm, with a mean of 1.474156 ± 0.251754 (2ULF); 0.69928–1.03576 mm, with a mean of 0.892738 ± 0.143129 (4ULF); 0.369483–0.61909 mm, with a mean of 0.507688 ± 0.10487 mm (BLF); 0.63811–0.9657 mm, with a mean of 0.827538 ± 0.13944 mm (TO).

In LF, R, the fracture displacements were as follows: 0.864508–1.3813 mm, with a mean of 1.148656 ± 0.215269 (2ULF); 0.34336–0.529722 mm, with a mean of 0.45333 ± 0.079228 (4ULF); 0.3058–0.52653 mm, with a mean of 0.427316 ± 0.09265 mm (BLF); 0.22933–0.40146 mm, with a mean of 0.329304 ± 0.072344 mm (TO).

In LF, L, the fracture displacements were as follows: 0.99393–1.51846 mm, with a mean of 1.278172 ± 0.218547 (2ULF); 0.74349–1.10267 mm, with a mean of 0.940034 ± 0.150294 (4ULF); 0.34472–0.57074 mm, with a mean of 0.468978 ± 0.094916 mm (BLF); 0.64191–0.95812 mm, with a mean of 0.818444 ± 0.132699 mm (TO).

In extension, the fracture displacements were as follows: 0.64968–0.99296 mm, with a mean of 0.838626 ± 0.143874 (2ULF); 0.49957–0.75405 mm, with a mean of 0.642018 ± 0.107391 (4ULF); 0.255165–0.42324 mm, with a mean of 0.348045 ± 0.07079 mm (BLF); 0.318113–0.50368 mm, with a mean of 0.423439 ± 0.078365 mm (TO).

In flexion, the fracture displacements were as follows: 1.20876–1.90684 mm, with a mean of 1.588204 ± 0.289955 (2ULF); 0.58728–0.87833 mm, with a mean of 0.751386 ± 0.12187 (4ULF); 0.39535–0.67403 mm, with a mean of 0.54825 ± 0.116783 mm (BLF); 0.55314–0.8559 mm, with a mean of 0.724312 ± 0.126621 mm (TO).

## 4 DISCUSSION

This FEA study aims to analyze the performance of the ULF in treating USF. It is widely acknowledged that BLF and TO provide strong biomechanical support, offering multiplanar stability and allowing for early full‐weight bearing activities post‐operatively. [21, 22, 23, 24] However, some surgeons proposed that ULF might be sufficient for unilateral sacral fractures. We have noticed that there are publications on the use of 4ULF for treating AO/OTA Type C1 and C2 pelvic fractures, and in these publications, it is stated that this structure can provide sufficient stability. [15] This is why our study includes the 4ULF model.

In principle, lumbar fixation can indeed be applied at either L4 or L5 levels. The choice between these levels often depends on several factors, including the anatomical and pathological conditions specific to each case. For instance, if the pedicles of L5 are compromised, it would be more practical and safer to utilize L4 for fixation. Additionally, in minimally invasive procedures, using L4 can be advantageous as it is easier to insert the longitudinal rod at this level due to the lordosis positioning L5 deeper within the soft tissue.

In our model, we opted to fixate only at the L5 vertebral body. This decision was guided by our intention to bridge and fuse as few segments as possible, aligning with our clinical practice, especially considering that we often deal with younger patients. Minimizing the extent of fixation helps in preserving the range of motion and reducing the potential for long‐term complications associated with more extensive spinal fusion.

Based on the mechanobiology of fracture healing, intermittent movement of the fracture ends (controlled micromovements between the fracture ends, which are important mechanical parameters in the fracture healing process) can promote callus formation and accelerate fracture healing. [25, 26] According to research, the optimal range for these micromovements is between 0.2 and 1 mm. Movements exceeding this range can have a negative effect on fracture healing [27, 28, 29].

Therefore, in this study, only internal fixation that maintains fracture displacement within the range of 0.2‐1 mm is considered to have “sufficient stability.” Considering the individual differences among patients and the complexity of real‐world scenarios (such as variations in weight or unexpected movements leading to increased load), we believe that displacements approaching the acceptable range's boundary also pose certain risks.

In our study, the 2ULF structure, comprising a single segment and a single iliac screw, is clearly unstable. In any posture, its fracture displacement far exceeds that of the other three models, which is evident in the box plots (Figure 9). Apart from the extension posture, the fracture displacement in all other postures is also greater than 1 mm, which is clinically unacceptable [30].

Regarding the 4ULF, although its average displacement value is less than 1 mm, this value is very close to 1 mm. Moreover, in RL and LF, L postures, the fracture displacement exceeds 1 mm. This indicates that patients with USF (Unilateral Sacral Fractures) under the 4ULF structure have a risk of experiencing unacceptable fracture displacement during full‐weight bearing activities. At the same time, we should note that there is a clear issue of stress concentration in the internal fixation of the 4ULF structure, primarily occurring at the L5 pedicle screw, specifically between the screw tail and the screw body. Interestingly, in the study that applied the 4ULF structure, a case of postoperative internal fixation failure was reported, with the failure occurring precisely at that position. Furthermore, although they stated in their article that the 4ULF structure could provide sufficient stability, they made more cautious requirements for postoperative mobilization of patients—gradually using a walking aid to stand and walk 3 weeks after surgery, which is consistent with our study results.

For the TO structure, although the overall fracture line is less than 1 mm, we observed that during RL and LF, L postures, the maximum fracture displacement reached up to 0.9657 and 0.95812 mm, which we consider to be quite risky. While there are numerous biomechanical experiments and clinical studies on the TO structure, most research involves simple loading of vertical forces or rotational forces, [31, 32, 33] with few studies simultaneously simulating and comparing all seven movements. Our study found that when the model is flexion to the left or rotated to the left, the maximum fracture displacements are close to 1 mm (0.95812 and 0.9657 mm), indicating that these movements pose a risk of fracture displacement. This risk is primarily associated with the unilateral TO structure comprising a single segment, a single iliac screw, and an S1 midline level sacroiliac screw. Existing literature reports that different sacroiliac screw lengths and fixation of different segments can affect stability. [34] However, literature has also demonstrated that for the TO structure, simultaneously fixing both L4 and L5 segments does not significantly improve the vertical stability of sacral fractures. Fixation of only the L5 segment does not fall short in terms of biomechanical effectiveness. [31] Nevertheless, we believe these two postures (RL and LF, L) are still the most unstable in the unilateral TO structure.

For the BLF structure, we can see that it performs relatively stable in all postures, which also demonstrates the advantages of BLF. Regardless of the posture, this structure effectively distributes the forces transmitted from the lumbar spine to the sacrum onto the ilium, thereby indirectly maintaining the stability of the sacrum. According to previous studies, this structure is also the most stable [35, 36].

We do not seek to negate the utility of ULF entirely. In reality, pelvic fractures are often complex and severe, and there may be extreme cases where bilateral fixation or sacroiliac screws are not feasible for surgeons. Indeed, ULF can provide a degree of stability for unilateral sacral fractures. However, in such scenarios, it may be necessary to extend the duration before allowing weight‐bearing postoperatively, with a focus on closely monitoring fracture healing, as practiced in the previously mentioned approach. Nonetheless, when opting for unilateral fixation, we strongly recommend enhancing the structure with an additional sacroiliac screw to convert it into a TO structure, where feasible. According to our study results, the inclusion of the sacroiliac screw in the TO structure markedly improves the stability of the fracture in various postures. This adaptation clearly shows a significant advantage in overall fracture stabilization, striking a better balance between minimally invasive approaches and effective treatment outcomes.

This study has certain limitations that should be considered. The employed finite element models primarily focused on the skeletal and ligament systems, excluding muscle forces, which is a common approach in similar finite element analyses. We utilized a singular lumbar‐pelvic model for our analyses to reduce the variability in bone and ligament properties. While our fracture models do not encompass all possible real‐life scenarios, they still offer valuable insights into unilateral sacral fractures (USF). It is important to note that this finite element study primarily assessed immediate postoperative stability, without delving into the long‐term biomechanical implications. Given these limitations, our conclusions would benefit from further validation through clinical retrospective studies and cadaveric biomechanical experiments to ascertain their practical applicability. Therefore, the application of our findings in clinical settings should be approached with caution.

## 5 CONCLUSIONS

In conclusion, our analysis indicates that the ULF structure with a single segment and a single iliac screw(2ULF) cannot provide sufficient stability; the ULF structure with double segments and double iliac screws(4ULF), although providing a degree of stability, still falls short compared to the traditional TO and BLF structures. We strongly advise physicians to be more cautious in considering the timing of weight‐bearing activities for patients undergoing such fixation to ensure safety; the 4ULF structure exhibits a significant issue of stress concentration at the L5 level; Furthermore, patients with unilateral TO fixation using a single segment and a single iliac screw should be cautious to avoid postures involving rotation or lateral flexion to the fracture side during postoperative weight‐bearing activities.

## AUTHOR CONTRIBUTIONS

Wolfgang Lehmann and Jie Yang contributed to research design; Jie Yang contributed to acquisition, analysis, and interpretation of data, and drafting the paper; Kai O. Böker, Xishan Li, and Xiang Zhou contributed to acquisition and interpretation of data, and revising of the paper. All authors have read and approved the final submitted manuscript.

## CONFLICTS OF INTEREST

The authors declare no conflicts of interest.
